# Control of nuclear organization by F-actin binding proteins

**DOI:** 10.1080/19491034.2016.1267093

**Published:** 2017-01-06

**Authors:** Karin Pfisterer, Asier Jayo, Maddy Parsons

**Affiliations:** a Randall Division of Cell and Molecular Biophysics, King's College London, New Hunts House, Guys Campus, London, UK; b Department of Basic Sciences, Physiology Unit, San Pablo CEU University, Monteprincipe Campus, Madrid, Spain

**Keywords:** actin, cell migration, Fascin, Nesprin-2, nuclear movement, nuclear deformation

## Abstract

The regulation of nuclear shape and deformability is a key factor in controlling diverse events from embryonic development to cancer cell metastasis, but the mechanisms governing this process are still unclear. Our recent study demonstrated an unexpected role for the F-actin bundling protein fascin in controlling nuclear plasticity through a direct interaction with Nesprin-2. Nesprin-2 is a component of the LINC complex that is known to couple the F-actin cytoskeleton to the nuclear envelope. We demonstrated that fascin, which is predominantly associated with peripheral F-actin rich filopodia, binds directly to Nesprin-2 at the nuclear envelope in a range of cell types. Depleting fascin or specifically blocking the fascin-Nesprin-2 complex leads to defects in nuclear polarization, movement and cell invasion. These studies reveal a novel role for an F-actin bundling protein in control of nuclear plasticity and underline the importance of defining nuclear-associated roles for F-actin binding proteins in future.

## Introduction

Cells have to dynamically adapt their cell shape to respond to changes in the extracellular environment. This is a key feature of cells in many physiologic and pathological situations, ranging from embryonic development and differentiation to wound healing and metastatic cancer cell migration. This requirement for shape adaptation is particularly challenging with respect to the manipulation of the nucleus, a rather rigid, large, enveloped intracellular compartment containing a densely packed network of chromosomes and associated proteins. The ability of cells to regulate the shape and position of the nucleus represents a key rate-limiting step in their movement through confined or complex environments.

## LINC-ing the nucleus

**Figure 1.**
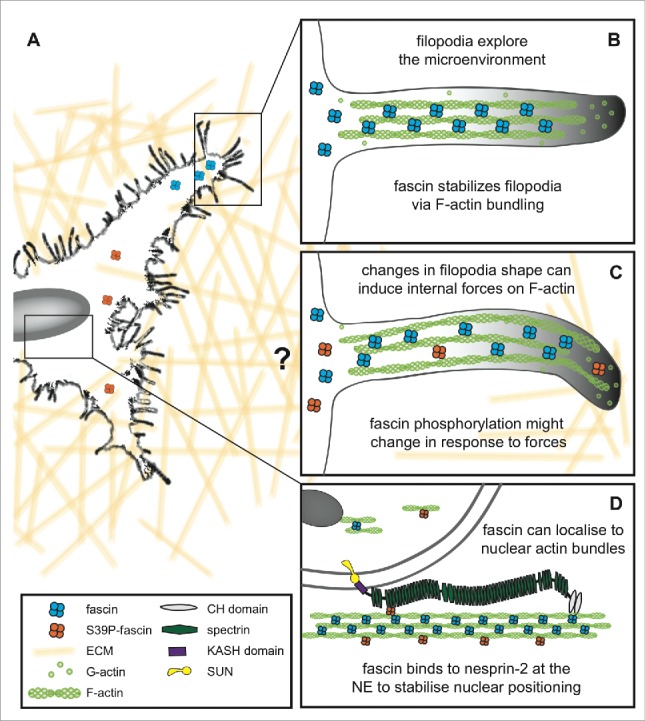
Fascin function at the cell periphery and NE/nucleus. (A) Migrating cells use finger-like protrusions called filopodia to explore the microenvironment. (B) Schematic view of a simplified filopodium where fascin, which is highly upregulated in tumor cells and cancer metastasis, is critical for F-actin bundling and filopodia stability. Fascin bundles F-actin by binding 2 adjacent filaments and thereby stabilizes filopodia. (C) Interaction of filopodia with the ECM can induce twisting and bending that exerts forces on actin filaments, potentially altering fascin phosphorylation status and thus F-actin bundling. (D) Forces and/or secreted factors can induce translocation of fascin to the nuclear periphery where it interacts with Nes2G, which is anchored at the outer NE via the KASH domain and interacts with SUN at the inner NE. Nes2G can directly interact with F-actin via its CH domain. Fascin stabilizes this interaction and thereby can efficiently regulate nuclear positioning and deformation during cell migration. Only the giant isoform of Nesprin-2 is shown in this simplified diagram. Fascin can also be shuttled into the nucleus and contributes to intra-nuclear actin filament bundling. CH, calponin homology; ECM, extracellular matrix; F-actin, filamentous actin; G-actin, globular actin; KASH, Klarsicht, ANC-1 and Syne homology; NE, nuclear envelope; Nes2G, Nesprin-2 Giant; P, phosphorylation; S39, Serine 39; SUN, Sad1p, UNC-84.

The nuclear envelope (NE) consists of a double lipid bilayer with an outer and inner membrane and allows both spatial separation and integrity of its content and exchange of proteins and genetic transcripts though nuclear pores with the cytoplasm. Mechanoresponses can be delivered to the nucleus by physically connecting the nucleoskeleton and the cytoskeleton via the well-conserved Linker of the Nucleoskeleton and Cytoskeleton (LINC) complex,^1^ which anchors the inner and outer NE to components of the cytoskeleton. A LINC-bound perinuclear ‘actin cap’ consist of ordered contractile actin filament bundles and also contribute to the nuclear shape in response to cellular shape changes.^2^ Actin cap fibers terminate at basal focal adhesions and act to transduce mechanical forces from the extracellular environment to the nucleus via LINC complex proteins.^3^ KASH (Klarsicht, ANC-1, and Syne homology) domain proteins, such as Nesprins, bind the outer nuclear membrane and interact with SUN (Sad1p, UNC-84) domain proteins in the inner nuclear membrane, such as SUN 1–5 and SPAG4 in vertebrates.^4^ Nesprins 1–4 are KASH domain proteins and recent studies have identified numerous isoforms of Nesprins-1 and −2 in mammals.^5^ Depending on size and isoform Nesprins have a varying amount of spectrin repeats (SR) forming a rod domain followed by the transmembrane and KASH domains.^5,6^ The so-called giant isoforms of Nesprins-1 and −2 have molecular weights exceeding 800 kDa, multiple SRs and have been shown to bind directly to F-actin via the N-terminal calponin homology (CH) domain.^5^ This interaction controls nuclear positioning and is vital for the polarization of migrating cells.^7^ We found recently that the F-actin-bundling protein fascin specifically interacts with the C-terminal region of Nesprin-2 at the NE allowing for increased connectivity between the nucleus and F-actin and facilitating nuclear deformability in response to environmental changes in migrating cells^8^ (Fig. 1D). This mechanism might be particularly vital for understanding cancer cell tissue invasion and extravasation from the primary tumor where cells have to squeeze through tight interstitial spaces. The regulation of nuclear plasticity to prevent DNA breakage has recently been an area of active interest. The formin FMN2 is essential in creating perinuclear adhesion actin fibers that protect the nucleus from rupture and DNA breakage during 3D cell migration. Interestingly, FMN2 acts independently of KASH family proteins. We observed that fascin perinuclear localization is dependent on KASH-domain proteins in *Drosophila*, which have also been shown to be crucial for TAN line formation.^9^ Transient NE rupture is induced through contractile actin fibers that increase pressure on the nucleus via LINC complex proteins^10^ and restoring the NE integrity requires components of the endosomal sorting complexes required for ESCRT-III machinery.^11^

The LINC complex consists of various members differentially expressed depending on cell type and tissue-specific heterogeneity has been shown to occur for the Nesprin 5′ UTR.^5^ As fascin is known to be highly upregulated in various tumor cell types, this suggests that tumor cells might specifically utilize elevated fascin levels to guarantee efficient cell migration. This would act to regulate both stability of cell protrusions such as filopodia to explore the environment (Fig. 1B) and also nuclear movement and deformation to promote invasion.

## Breaking the LINC

Proteins of the LINC complex are essential players in the control of rearward nuclear movement and reorientation during cell polarization.^9^ Nesprins are vital for the maintenance of the NE integrity and architecture. Mutations in genes encoding Nesprins have been linked to several diseases. Dysfunctional Nesprin-1/2 can result in general myopathies and cardiomyopathies, and in combination with mutations in SUN1 causes Emery Dreifuss muscular dystrophy.^12^ Interestingly, mutations in Nesprins could be connected to high-frequency hearing loss (Nesprin-4), visual impairment (Nesprin-2), bipolar disorder, depression and autism.^7^ Screening for DNA variations in Emery Dreifuss patients showed that Nesprin-1 and −2 were often mutated and nuclei were deformed in patient fibroblasts.^13^ *In vivo* experiments using Nesprin-2 knockout mice revealed that skin fibroblasts and keratinocytes developed giant nuclei^14^ providing further evidence for Nesprins as critical regulators of nuclear shape and function.

Mutations in Nesprin-1 and −2 have also been associated with several types of cancers, like ovarian, breast, lung and colorectal cancer and gastrointestinal stromal tumor.^12^ However, mechanisms linking nuclear deformation or movement during invasion have not yet been clearly defined. Interestingly, fascin is highly upregulated in all types of cancer studied to date. We found that specifically uncoupling fascin-Nesprin interactions without altering filopodia-associated fascin reduced the migration and invasiveness of cancer cells,^8^ suggesting an important role for NE-associated fascin in control of cancer cell motility. Fascin is also well-known as to promote cancer cell migration through its well characterized F-actin bundling role within filopodia.^15-17^ Further studies will be important to define whether fascin overexpression in the absence of Nesprin or the presence of fully functional, mutated and/or truncated forms might predict cancer invasiveness and metastasis formation. However, given the enormous biologic heterogeneity across cancer types, this also highlights the potential for new personalized therapy strategies according to the presence or absence of these cancer biomarker combinations. Ideally, optimized therapeutic targeting of fascin could attack cancer cells from 2 different angles: through elimination of filopodia as well as disrupting the cells ability to navigate through changing extracellular environments.

## Links to the LINC: Emerging additional cytoskeletal adaptors

Nesprin-1 and −2 isoforms that contain CH domains can make direct links to the actin cytoskeleton to provide scaffolding between the NE and the cytoskeleton. We have shown that fascin physically engages in this interaction by further cross-linking F-actin and the LINC complex protein Nesprin-2, which may act to stabilize the connection of the nucleus to the cytoskeleton, as well as act as a sensor for changes in external forces to provide more rapid means to respond to extracellular environments (Fig. 1D). Interestingly, FHOD1, a diaphanous related formin, has also been recently shown to directly bind to Nesprin-2 (Nes2G), but in contrast to fascin, which binds at the C-terminus, FHOD1 binds the SR 10–13 closer to the N-terminus.^18^ Knockdown of FHOD1 did not disrupt F-actin retrograde flow, but instead loosened the connection of the nucleus to the actin cytoskeleton suggesting distinct roles for these actin-associated proteins in this context. Nesprin-2 and SUN2 build transmembrane actin-associated nuclear (TAN) lines^19^ along the nuclear membrane that allow tight coupling to F-actin. Whether TAN lines are distinct from actin caps in this context remains unclear. However, FHOD1 knock-down diminishes TAN line formation and results in aberrant nuclear movement.^18^ Although FHOD seems to provide an additional anchor for Nes2G to the actin cytoskeleton to enable TAN line assembly, FHOD1 expression does not impact on nuclear localization of fascin, nor its binding to Nesprin-2, suggesting these 2 Nesprin-2 binding partners are independently regulated. It therefore remains unclear whether these 2 F-actin linker proteins associate with Nesprin simultaneously or are required individually in different contexts. Elucidation of the upstream signals controlling the kinetics of these associations in a wider variety of cell types will provide significant insight into the environmental triggers or conditions that dictate Nesprin binding to fascin or FHOD1.

The C-terminus proximal spectrin-rich region of Nesprin-1/2 not only contains a binding site for fascin, but also distinct binding sites for further actin-associated proteins.^7^ Recently, BIN1 (also known as amphiphysin-2) has been reported to bind Nesprin-2 and the microtubule plus-end-binding protein CLIP170, which connects the nucleus to both the actin cytoskeleton and microtubules and promotes optimal nuclear positioning and shape.^20^ The inner nuclear membrane anchored protein emerin has been shown to interact with the last 2 spectrin-rich regions of Nesprin-1/2,^21^ although the purpose of this interaction has not yet been clarified. This does however suggest that emerin also localizes to the outer NE, which possibly allows it to act as a mechanosensor. Application of mechanical forces to isolated nuclei across Nesprin-1 resulted in tyrosine phosphorylation of emerin and subsequent nuclear stiffening.^22^ Recent reports indicate a possible role of mechanical forces in gene expression and in regulating signal transduction cascades in a range of cell types. Stress induced through compression, tension and shear forces can activate the Wnt/ß-catenin pathway.^23^ Interestingly, ß-catenin, an integral E-cadherin cell-cell adhesion adaptor protein and transcriptional co-factor, is increased in the nucleus upon shear force application.^23^ Emerin has been shown to shuttle α-catenin, which can interact with ß-catenin and Nesprin-2, to the nucleus. Conversely, nuclear accumulation of ß-catenin is restricted by emerin,^24,25^ suggesting a potential role for LINC complex proteins in acting as gatekeepers of transcriptional signaling. Cytoplasmic ß-catenin levels are increased upon Wnt pathway activation, which allows the formation of a quaternary complex of Nesprin-2, emerin, α- and ß-catenin at the NE and subsequent nuclear translocation of ß-catenin. Loss of Nesprin-2 reduces ß-catenin levels in the nucleus, possibly due to inefficient complex formation at the NE.^26^ Interestingly, fascin promoter activity and protein expression can be induced by Wnt/ß-catenin signaling and high fascin levels can be found at the invasive front of colon cancer tissue.^27^ As NE localized fascin is crucial to maintain the nuclear morphology and allow nuclear deformability,^8^ this step could be a molecular switch in solid tumors to increase cell invasion and metastasis formation through regulating nuclear plasticity.

## Nuclear envelope anchoring and nuclear actin: Potential co-regulation?

The suggestion that proteins at the NE may impact on nuclear shuttling through association with cytoplasmic cytoskeletal elements also raises the possibility of roles in organizing nucleoskeleton architecture. Of note in this context, fascin has recently been shown not only to localize to the nuclear periphery, but is also present within the nucleus itself (Fig. 1D). Fascin localizes to the nucleus both in mammalian cells, and during late-stages of *Drosophila* follicle development, and this nuclear translocation depends on prostaglandins signaling.^28^ The role and regulation of fascin within the nucleus is not yet clear, but the nucleus contains high levels of actin, both monomeric and filamentous forms,^29^ with actin filaments being shorter in length than those found in the cytoplasm. Fascin is required for endogenous nuclear actin bundles to form and depletion of fascin from *Drosophila* nurse cells increases the size and number of nucleoli suggesting a role in maintaining nuclear actin organization and compartments.^28^ Several other actin-binding proteins, whose function has been extensively investigated in the cytoplasm, are also present in the nucleus. These include cofilin (suggested to be important for actin monomer import and accumulation in the nucleus) and profilin (partly responsible for actin nuclear export).^30^ The nucleolus is not only a site of rRNA processing and synthesis. Recent studies indicate a role for nucleoli beyond ribosome biogenesis, such as cell cycle progression, stress response, senescence, apoptosis and cancer.^31^ High levels of fascin are only present in certain stages of *Drosophila* follicle development and active nuclear export lowers nuclear fascin levels and increases perinuclear fascin.^28^ It is therefore plausible that fascin import and export into and out of the nucleus is also tightly regulated in mammalian cells depending on cell cycle, stress or environmental influences. An active exclusion of fascin from the nucleus in cancer cells could be one mechanism to promote abundant fascin levels at the NE and in filopodia to enable efficient invasion. Mechanical forces exerted on cells from the extracellular matrix are rapidly transmitted via adhesion receptors to the cytoskeleton and subsequently to the nucleus. The shuttling of fascin between the NE and nucleus in response to changes in local tension may represent one strategy for cells to rapidly adapt to extracellular cues and re-position key actin-binding proteins to appropriate subcellular compartments. Further investigation of the regulatory signals that control such dynamic movements will be important to shed light on this important and conserved phenomenon.

## Intracellular shuttling: Regulating fascin localization and function

Fascin is made up of 4 β-trefoil repeats separated by short flexible linker regions^32^ and has 2 major binding sites for actin, one within the ßt1-ßt2 domains and the second within ßt3-ßt4, that combine to allow efficient F-actin bundling in filopodia.^32,33^ Serine 39 (S39) on ßt1 is an important regulatory site for the actin-bundling activity of fascin as its phosphorylation by PKCα inhibits actin bundling via the N-terminal domain^34^ and also promotes fascin binding to Nesprin-2 at the NE.^8^ This suggests that dynamic translocation of fascin occurs within the cell (Fig. 1), but the time scales and regulation of this are currently unknown.^34^ Filopodia contain bundles of F-actin stabilized by fascin, and act to explore the environment. Integrin binding to specific extracellular matrix cues results in increased PKC activation, fascin phosphorylation at S39 and subsequent loss of F-actin bundling resulting in more diffuse cytoplasmic distribution of fascin.^34^ It is possible that this may promote translocation of fascin to the perinuclear area and promote Nesprin-2 binding for the regulation of nuclear movement, plasticity and deformation during cell movement through 3D environments. Fascin binds to Nesprin-2 (SR51–53) in a highly conserved region, adjacent to the emerin binding site, suggesting a comparable functional role as mechanosensor. Crystal structures of fascin suggest a globular conformation with 2 binding sites for F-actin, one on either side.^33^ Lateral forces applied across the molecule upon F-actin-binding at one side and Nesprin-binding at the other might feasibly result in a conformational change in fascin and allow the accessibility of kinases or phosphatases. Whether fascin phosphorylation at serine 39 is altered upon nuclear force induction leading to enhanced binding to Nesprin-2 remains to be clarified.

Very little is known about how fascin is relocated throughout the cell, or if this is active transport or passive diffusion. The recycling endosomal protein Rab35, a member of the Rab family of GTPases, has been proposed as one potential regulatory protein involved in transporting fascin to the cell periphery in *Drosophila* and mammalian cells.^35^ In fibroblasts Rab35 is enriched near the plasma membrane and colocalises with fascin in filopodia, microspikes and lamellipodia. Overexpression of dominant-negative Rab35 limits the presence of fascin at the plasma membrane and increases cytoplasmic accumulation.^35^ However, overexpression of fascin can overcome this phenotype, suggesting a disruption of this spatial regulatory mechanism may occur in cancer cells. It also seems likely that other post-translational modifications may occur on fascin that lead to associations with as yet unidentified binding partners to control traffic and localization. Advances in more sensitive proteomics analysis methods may provide means to begin to test this possibility in future.

As fascin is highly upregulated in many tumor types, it is also possible that molecules that are secreted by the tumor microenvironment and favor tumor development and progression may play a role in regulating fascin association with F-actin at different subcellular sites. One example is Transforming Growth Factor ß (TGF-ß), a cytokine that is secreted by the tumor microenvironment, increases fascin promoter activity and expression levels via phosphorylation of the Smad3 linker region.^36^ Prostaglandins are transient bioactive lipids that are also often misregulated in cancer and can influence the adhesive, migratory and invasive potential of cancer cells.^37,38^ Prostaglandins have the potential to regulate the translocation of fascin into and out of the nucleus,^28^ which might represent another important mechanism to control fascin-dependent behavior in tumors. While we have much still to learn about the precise regulatory mechanisms in different contexts, what is clear is that fascin can perform multiple roles as an F-actin binding protein (highlighted in Fig. 1) to control different phenotypic endpoints. This opens up the possibility of alternative roles for other actin-regulatory proteins either at the NE or within the nucleus. Recent improvements in super-resolution, single molecule and correlative-light electron microscopy methods should provide ideal platforms to begin to dissect the precise subcellular dynamics and fate of these molecules in live cells in response to defined extracellular cues.

## Conclusion and future perspectives

Increasing evidence suggests that F-actin binding proteins that are classically associated with the assembly of peripheral, dynamic or architectural regulating cytoskeletal structures are able to associate with the NE and in the nucleus itself. This potential for molecular multi-tasking opens up new questions about how these F-actin binding proteins are spatio-temporally controlled to perform the correct function at the required location at the right time. Given the large number of Nesprin-1/2 isoforms being uncovered in specific tissues, it would also be interesting to determine whether other actin-binding proteins can co-associate with Nesprins in an isoform-specific manner. This may be particularly important in those isoforms where the Nesprin CH domains are absent as these proteins may provide essential links to the cytoskeleton. As many of these actin-associated molecules, including fascin, are implicated in the progression of several diseases, understanding these regulatory mechanisms in more detail will likely inform on future therapeutic strategies.
